# The effectiveness of stress-management-based cognitive-behavioral treatments on anxiety sensitivity, positive and negative affect and hope

**DOI:** 10.1051/bmdcn/2018080423

**Published:** 2018-11-26

**Authors:** Sara Sahranavard, Aliakbar Esmaeili, Reza Dastjerdi, Hamid Salehiniya

**Affiliations:** 1 Department of Psychology, Faculty of Medicine, Birjand University of Medical Science Birjand Iran; 2 Social Determinants of Health Research Center, Faculty of Health, Birjand University of Medical Sciences Birjand Iran; 3 Associate Professor of Psychiatry, Social Determinants of Health Research Center, Birjand University of Medical Sciences Birjand Iran; 4 Birjand University of Medical Sciences Birjand Iran; 5 Iran University of Medical Sciences Tehran Iran; 6 Department of Epidemiology and Biostatistics, Tehran University of Medical Sciences Tehran Iran

**Keywords:** Cognitive behavioral treatment, Anxiety sensitivity, Hope, Positive and negative affect

## Abstract

Background and objective: Anxiety sensitivity, positive and negative affection and hope are the important factors in promoting mental health of students. The purpose of this study was to investigate the effectiveness of stress-management-based cognitive-behavioral treatments on anxiety sensitivity, hope, positive and negative affect in female students of Medical Sciences.

Materials and methods: This research was a trail study with pre-test, post-test and control group. A sample of 30 subjects, were selected by available sampling and were randomly assigned using Block Randomization Method of two groups (experimental and control groups). Schneider’s hope questionnaire, Watson’s positive and negative affect questionnaire, Clarke and Tolgman’s questionnaire, Reiss *et al.’s* anxiety sensitivity of the revised index questionnaire, were completed in two stages (pre-test and *post-test)* by all subjects. A 6-session protocol of cognitive-behavioral group treatment was performed only on the experimental group. The data were analyzed using ANOVA and MANOVA analysis of variance.

Results: Two experimental and control groups with the mean 22, standard deviationl. 13, average age is 22 years. Stress-management-based cognitive-behavioral treatments were effective on the level of anxiety sensitivity and hope *(p* <0.016), however, it had no significant positive effect on the amount of positive and negative affect (*p* <0.016).

Conclusion: According to the results, it can be concluded that cognitive-behavioral treatments are effective on anxiety sensitivity and hope. Therefore, stress-management-based cognitive-behavioral training can reduce students’ anxiety sensitivity and increase their hopes for coping with challenges.

## 1. Background

Student years, especially at the University of Medical Sciences, is a timepiece that, the students are involved in a variety of subjects, such as their work environment (school, hospital, *etc.*), dwelling environments, and away from the family. These topics have different effects on their physical and mental health. One of the most common psychological problems is anxiety problems. Anxiety alone, as an evident symptom and with other disorders, forms the main core and the center for many psychiatric disorders [1].

The anxiety sensitivity is one of the major issues in the study history of anxiety disorders [2]. Anxiety sensitivity is the fear of anxiety symptoms that interprets the physical, psychological and social consequences as harmful and dangerous issues [3, 4]. Reminding stressful living problems causes anxiety sensitivity in students. Around, including parents, have a significant role in reducing and increasing support sources of students [5–7]. In general, the prevalence of this disorder in women is twice that of men. A lot of research suggests that people’s mental health has dropped off after starting school and this is still evident in the years of study. In fact, it can be said that medical students are more exposed to professional and academic stresses than other people in the community and students of other fields. The prevalence of anxiety modes and sensitivity among medical students compared to other students can be due to the large volume of books and literature studied in this field, the stress caused by numerous exams and the competitive environment of medical schools, which lead students to higher scores [1]. Also, in higher education levels, factors such as entering the hospital environment, dealing with patients, changing bed hours, participating in morning reports, dealing with other medical personnel, and initiating cooperation with professors, residents and the like can play a more effective role in increasing and exacerbating the anxiety sensitivity of individuals [8]. In past studies, the effect of gender on anxiety among medical students has been well indicated. Despite the fact that female students have demonstrated good competence in clinical competence and skills in dealing with their patients, reports indicate a higher prevalence of anxiety and anxiety sensitivity among female students [9]. This can lead to devastating effects on understanding the capabilities of female students from both themselves and from the perspective of others and affect the performance and capabilities of this group. Research shows that emotional states and expressions vary in different cultures. The results showed a significant relationship between academic achievement, anxiety and depression [7, 10]. Also, the results of other research showed that conditioned students compared with unconditioned students had significantly more symptoms in mental health such as obsession, depression, hostility, anxiety, phobia, psychosis and eating disorders and have lower mental health [7, 11]. Problems such as anxiety, depression, disturbances between student parents and poor communication between the student and parents can have a negative effect on a large number of coping factors such as personal and educational consistency, mental health, and social support [7]. Of course, having high anxiety sensitivity alone cannot be so problematic [12]. Also, as long as people with high anxiety sensitivity accept emotionally disturbing thoughts, they are likely to be able to inhibit their increased anxiety experiences [13].

Among the concepts related to anxiety sensitivity, hope and positive and negative affect can be mentioned. The beneficial effects of these variables on the physical and mental health have been confirmed in various researches [14]. After presenting the theory of hope and creating a tool for measuring it by Schneider, many studies looked at the relationship between hope and various variables of mental and physical health [15, 16]. Hope is a fundamental concept in religion, marriage, mental health and counseling and psychotherapy. Hope is one of the topics of positive psychology [17] and is one of the most important variables which has a powerful effect on reducing anxiety sensitivity and development of mental health [15, 18] and prevents the perception of vulnerability and anxiety disorders [15, 19].

Hope suggests that the goals can be achieved in life. People who have high hopes are more capable of solving social problems and they use less negative coping measures (such as retiring from the crowd, avoiding problems). There are many ways to achieve the goal. Recognition of hope creates positive emotions and psychological well-being in individuals. This issue has a positive correlation with variables such as optimism, self-efficacy, selfesteem and health [20], well-being of performance, coping strategies and emotional regulation [15, 18], satisfaction and positive emotions [21], positive affect [19] and self-worth [22]. On the other hand, the hope variable is negatively correlated with depression [16, 22–27], anxiety, anxiety sensitivity [24, 27], exhaustion [16, 26], stressful life events [28], and in general with negative emotions. However, some studies have shown that people who had high hopes, have lower satisfaction of life and vice versa [29].

Some scientists like Darwin emphasize this point that affections (positive and negative) are beneficial to humans because targets their activities and forces humans to do useful things [30]. Affection and moods are among the variables that contribute to the incidence of mental disorders. In terms of nature, the positive and negative affect of two phenomena are relatively independent and separate from one another [31]. He negative affect is a kind of inner grief that means to the extent that the person has unpleasant feelings such as anger, grief, hatred, humiliation, guilt and fear [32, 33]. On the other hand, positive affect is a state of active energy, high concentration and enjoyable employment which includes positive mood states such as joy, empathy, enthusiasm, interest and self-esteem. The results of various researches show that having a person’s enjoyable communication with the environment creates the states of being active, enthusiastic and hopeful, successful, and reduces the anxiety sensitivity of the emotions and positive emotions [34]. People with high positive affect are energetic and spirited and enjoy life. In contrast, those with a high negative affect are anxious, worried, and energy-poor. Martin has shown that positive affection has been linked to the establishment of broad social relationships, assistance behavior, accuracy, focus, and high decision-making ability [35]. Positive affect contributes to improving the physical health of the person by strengthening the immune system. In mental health, studies have shown that positive affect can counteract negative affect and their destructive effects [36].

## 2. Objective

The purpose of his study was to evaluate the effectiveness of group training of CBT-based stress management on anxiety, hardiness and self-efficacy in female students of Birjand University of Medical Sciences.

## 3. Materials and methods

### 3.1 Subjects

This research was a trail study with pre-test, post-test and control group. Samples were available chosen. The population fully consists of female students of Birjand University of Medical Sciences in Iran, a sample of 30 subjects, were selected by available sampling, were selected by available sampling, and participants were randomly assigned using Block Randomization Method of two groups.

The inclusion criteria consisted of gaining a higher score than the average in the anxiety inventory, living in dormitory, being informed and satisfaction. Thus, unsatisfied students, the graduate ones, those who didn’t live in dormitory, and students with lower score than average in the anxiety inventory were excluded from the sample. The data were analyzed using the mean and standard deviation at the descriptive level and the ANOVA and MANOVA tests at the inferential level.

### 3.2 Intervention

Patients were cognitive behavioral treated group-based for 6 sessions of 90 minutes and twice a week by a clinical psychologist. Before the intervention, a pre-test was performed for the experimental and control groups and after that, the interventions were held for 6 sessions and at the end, a post-test was performed. The contents of the sessions are as described in Table 1. During this time, the control group did not receive any training and was on the waiting list. One week after experimental training, all individuals in both experimental and control groups completed the inventories.

**Table 1:** The number and content of the sessions.

Session	Content of the sessions
1	Awareness of stress and its coping ways: Self-awareness
2	Do not be indifferent to stress: Mental methods
3	Adapt to life: Physical methods of coping with stress
4	Study skills, exam preparation and time management
5	Group power: Interpersonal relations skills
6	Treat yourself to merit: cultivate self-esteem and honor, prevent depression and anxiety and deal effectively with them

### 3.3 Procedure

The study was approved by the Research Ethics Committee of the Birjand University of Medical Sciences (Birjand, Iran). The research was carried out after the consent form was signed by the students. All Procedure was conducted according to the declaration of Helsinki.

### 3.4 Measures

#### 3.4.1 Anxiety sensitivity

Anxiety sensitivity questionnaire was developed by Reise *et al.* [37, 38] This questionnaire is a self-reporting tool with 16 questions which is graded according to the five-point Likert scale (very low = 0 to very high = 4). The degree of experience of fear of anxiety symptoms with higher scores is determined. Scores range from 0 to 64 [39]. The structure of this questionnaire consists of three factors of fear of future concerns (8 questions), fear of lack of cognitive control (4 questions) and fear of observing anxiety by others (4 questions) [40]. The psychometric properties of this scale show its high internal stability (alpha ranging from 0.80 to 0.90). The test-retest reliability of this questionnaire after two weeks and three years was 0.75 and 0.71 respectively which indicates that, anxiety sensitivity is a persistent personality issue [37]. The validity of this questionnaire has been calculated in Iranian sample based on three methods of internal consistency, test-retest and split-half analysis. For all scales, the credit coefficients were 0.93, 0.97 and 0.95, respectively. Validity was calculated based on three methods of simultaneous validity, correlation of subscales with total scale and also with each other and factor analysis. Simultaneous validity was performed through the implementation with the questionnaire with a correlation coefficient of 0.56. The correlation coefficients with the total score was in satisfactory range and fluctuated between 0.74 and 0.88 [41]. The reliability of the anxiety sensitivity scale in this study was 0.90 using Cronbach’s alpha coefficient test.

#### 3.4.2 Positive and negative affect

This questionnaire was prepared and presented by Watson, Clarke *et al.* [34] In this tool, 20 concepts which expresses the feeling (10 positive feelings and 10 negative feelings) were graded based on 5-point Likert scale (1 = very low to 5 = very high). The validity and reliability of this scale were confirmed in various studies. Abolqasemi obtained the coefficient of internal correlation of components and total scale between 0.74 to 0.94 and its coefficient of reliability 0.65, respectively. All of them were significant (*p* <0.01), indicating the validity of the structure of this scale. The validity of this tool has been met by calculating the correlation between positive and negative affect with some research tools that measures the structures related to these two scales. For example, positive affect correlation with the Hapkins score check list of 0.72 and positive affect correlation with the apparent anxiety scale of 0.35 have been reported [34] The reliability of the positive and negative affect scale was 0.91 by Cronbach’s alpha coefficient test.

#### 3.4.3 Hope

The 12-point scale was designed by Schneider (16) for ages 15 and over and includes two subscales of the passage and motivation. It takes a short time (2 to 5 minutes) to respond. Each question is a continuum of 1 (completely false) to 4 (perfectly correct). In order to study the validity and reliability of this questionnaire, 60 students of Isfahan University were selected. The results indicate that the questionnaire has an internal consistency of 68%. Also, there was a significant correlation between this questionnaire and positive affect (r = 46%, p = 34%) and optimism (r = 64%, p = 0.001) indicating simultaneous validity. Reliability of the Hope scale in this study was 0.91 obtained by Cronbach’s alpha coefficient test.

## 4. Results

Table 2 shows the mean, standard deviation, minimum and maximum age of the experimental and control groups. In the both groups, the average age is 22 years whereas the age varies from 20 to 24 years. Meanwhile, the both groups were matched in the age variable.

**Table 2:** The mean, standard deviation (SD), minimum (min) and maximum (max) age of the experimental and control groups.

Max	Min	SD	Mean	Number	Group	Variable
24	20	1.13	22.00	15	experimental	Age
24	20	1.13	22.00	15	control	

The mean and standard deviation of the main variables of the study are presented in Table 3 for the experimental and control groups. As can be seen, for the experimental group in the posttest, the mean of anxiety decreased whereas the mean of hardiness and self-efficacy increased.

**Table 3:** The mean and standard deviation (SD) of the main variables of the study for the experimental and control.

SD	Mean	Measure stage	Group	Variable
22.93	36.00	pre-test	experimental	Anxiety sensitivity
10.30	13.60	post-test		
22.68	35.93	pre-test	control	
22.68	33.53	post-test		
9.81	71.33	pre-test	experimental	hop
6.11	76.93	post-test		
9.81	711.33	pre-test	control	
10.31	70.46	post-test		
8.17	58.80	pre-test	experimental	Positive and negative affect
7.81	60.40	post-test		
8.17	58.80	pre-test	Control	
8.72	58.20	post-test		

The Kolmogorov-Smirnov test was used to evaluate the assumption of normality of the variables. The results of this test show that according to the significance level, all variables follow the assumption of normality *(p* > 0.05).

The assumption of the homogeneity of regression slopes means that the regression coefficient of the dependent variable has the same coefficient of the covariance variables in the groups. To test this assumption for each of the variables, analysis of variance (ANOVA) F-test was used. In according to obtained results (Table 4), the regression coefficient, F, that calculated for group interaction and the pre-test are not significant *(p* > 0.05). As a result, there is no significant difference between the coefficients and hence the assumption of homogeneity of regression coefficients is confirmed.

**Table 4:** The results of ANOVA F-test.

Sig.	F	df	sum of squares	Statistical index (SI)	Variable
0.26	2.16	1	4.66		Anxiety sensitivity
0.23	1.11	1	8.41	interaction between group and pre-test	Hop
0.18	1.81	1	12.07		Positive and negative affect

### 4.1 Analysis of the assumptions

Data analysis for multivariate analysis of variance (MANOVA) shows that the assumptions of independence, normality, homogeneity of variances, homogeneity of variance-covariance matrix and homogeneity of regression slope for performing parametric tests are established. Regarding the assumptions made in our study, it can be concluded that the data of this research have the ability to enter the multivariate covariance analysis; therefore we can investigate the differences between the dependent variables of two groups. The results of multivariate analysis of covariance (MANCOVA) of post-test scores in the experimental and control groups are summarized in Table 5. As can be seen, there is a significant effect for the cognitive therapy (CT) (independent variable) after the elimination of the pre-test impact. Therefore there is a significant difference between at least one of the dependent variables in the experimental group with the control group (Wilks’ lambda = 18.38*,p* < 0.001).

**Table 5:** The results of multivariate analysis of covariance (MANOVA) of post-test scores in the experimental and control.

Sig.	Error df	Hypothesis df	F	Value	Test type	Statistical index
0.001	23.000	3.00	11.03	0.59	Pillai’s Trace	
0.001	23.000	3.00	11.03	0.41	Wilks’ Lambada	The difference between two groups by controlling the pre-test effect
0.001	23.000	3.00	11.03	1.44	Hotelling’s Trace	
0.001	23.000	3.00	11.03	1.44	Roy’s Largest Root	

To evaluate the effect of independent variable on dependent variables, the results of one-way covariance analysis in MANCO- VA context are listed in Table 6, whereas the effect size is equal to the effect of the independent variable on the dependent variable and the power of test indicates the adequacy of the sample size. It is worth noting that the significance level achieved for research variables is smaller than the significance level of 0.016 obtained from Bonferroni correction for multivariate covariance analysis (dividing the significance level of 0.05 by 3 dependent variables). Based on the obtained means, it can be concluded that cognitive therapy (CT) is effective on the level of anxiety, hardiness and self-efficacy of students, therefore, the research hypothesis is confirmed.

**Table 6:** The results of one-way covariance analysis in MANOVA.

Power of test	Effect size	Sig.	F	df	sum of squares	Source of variation	Variables
0.97	0.39	0.001	19.08	1	2990.18		Anxiety sensitivity
0.82	0.26	0.003	10.63	1	313.46	between-group	Hop
0.57	0.16	0.035	4.96	1	36.27		Positive and negative affect

## 5. Discussion

The effectiveness of stress-management-based cognitive-behavioral treatment on anxiety sensitivity, hope, positive and negative affect in female students of Medical Sciences was studied. In the first hypothesis of the study, the effectiveness of stress-management-based cognitive-behavioral treatment on anxiety sensitivity was studied. The results showed that these interventions had an effect on the symptoms of anxiety sensitivity and decreased symptoms. The results of this study were consistent with some findings [42, 43]. Anxiety sensitivity in most researches has been considered as a special variable in the experience of severe anxiety and anxiety disorders [44, 45].

As previously mentioned, anxiety sensitivity is defined as fear of signs of physical symptoms of anxiety and the expectation that these symptoms will lead to annoying consequences. Fear of physical symptoms can lead to excessive rust and internal selfmonitoring of signs and internal feelings. Therefore, fear of anxiety arousal and symptoms of sympathetic system, such as sweating, breathing and increasing heart rate, are high in people with anxiety sensitivity. Therefore, there is a defective cycle between physical emotions, evaluations and negative interpretations which permanently and persistently puts the person in vigilance state about physical symptoms associated with anxiety. High levels of anxiety sensitivity may be associated with increased rumination related to the catastrophic topic of anxiety related issues. Accordingly, research shows that worry is associated with decreased activity of the parasympathetic system and cardiovascular arousal.

New evidence of anxiety and anxiety sensation show that, psychological factors, especially, hope, play an important role in anxiety sensitivity and along with emotional regulation, hope constitutes the foundation of many psychological issues.

Another study showed that stress-management cognitive- behavioral treatment is effective in increasing the hope of female students as; post-test scores of hope of students were significantly increased.

Increasing the level of hope has a significant relationship with reducing mental health problems. Hope and positive attitude toward the future in students can moderate the harmful effects of student anxiety sensitivities and make more adaptive emotional responses. Students who have higher hope in coping with mental health, especially anxiety sensitivity, have better coping skills and less use of denial. These students are more humorous, less involved with their emotions (both positive and negative) and they use more problem-oriented coping methods to solve their problems. Hopeful students are less involved with common educational problems such as distraction, lack of social skills, helplessness, and disproportionate behaviors [46, 47].

The result, as well as Petmigel 2000, show that hope is related to academic performance, so that higher level of hope increases student’s average score. Findings related to the relationship between hope and mental health showed that there is a significant relationship between these two variables. This conclusion is consistent with the findings of others [48–50].

Hope is the emotional force that drives imagination toward positive things and gives humans energy, vitality, and ability to get rid of problems. Students who have hope achieve what they deserve and try for, have less worry and anxiety sensitivity symptoms. These people become less involved with their emotions when their anxiety tends to rise. Hopeful people usually view the world as a safe and promising place and they are trying hard to achieve what they want. On the contrary, those who think of the world as insecure and anxious, they may resort to this belief irrational that, whatever I try and hope, I will not get it. These people, in addition to the pressure that others impose on them, they are passive in the face of accidents, and may have aggressive behaviors to restore their rights. These people may experience signs of anxiety and frustration symptoms. Therefore, one can expect that with hope, the amount of anxiety sensitivity will decrease dramatically [51–53].

Regarding the role of gender in increasing hope and reducing anxiety sensitivity, it can be said that the sources of hope and happiness vary from country to country for both genders, men are more likely to be encouraged by jobs, economic satisfaction, *etc*., and women by children, educational success, family health, and so on. Hope in female students can be higher and better than male students [27].

On the other hand, the results of this study indicate that, cognitive-behavioral treatment do not affect positive and negative affect. To analyze this result, it can be inferred that, on the one hand, the students of the University of Medical Sciences, due to the difficult conditions of admission and the training process at the university and on the other hand, they have higher ability to manage their excitement as they learn specialized training during internship. Among other factors effective in obtaining this result, there are different characters, cultures and subcultures among students in this research which are considered to be hidden and intervening variables in this research. In the end, we should point out that other psychological treatments, such as acceptance-based treatment, Intensive Short Term Dynamic Psychotherapy)IST- DP(, individual treatment, etc., may affect the students’ positive and negative affect [15, 16].

## 6. Conclusion

In this research, the findings and suggestions of previous research have been developed based on new variables. It is suggested that special attention be paid to students’ feelings and emotions alongside educational issues. Educational practitioners should seek to improve emotional issues which enhances students’ thinking and prepares them to cope with class challenges. This gradually increases the student’s academic skills and as a result, they expect more education and academic success.

## 7. Limitation

The limitations of this study include lack of cooperation in order to follow up patients and personal and emotional variables were not controlled. Also the student’s homework was not controlled by the researchers.

## Consent to participate

All students signed the informed consent form to participate in the study, following all the necessary ethical recommendations inherent to a project developed with humans.

## Funding statement

The authors received no specific funding for this work.

## Conflict of interest statement

The authors disclose no conflicts of interest.
